# Discovering aspects of health—experiences of a web‐based health diary among adults with primary immunodeficiency

**DOI:** 10.1002/nop2.182

**Published:** 2018-07-10

**Authors:** Christina Petersson, Janne Björkander, Ramona Fust

**Affiliations:** ^1^ Department of Nursing at School of Health and Welfare, Jönköping University and Member of IMPROVE Research Group at the Academy for Improvement of Health and Welfare Jönköping University Sweden; ^2^ Academy for Health and Care Futurum Jönköping Sweden; ^3^ Department of Clinical Immunology University Hospital of Linköping Linköping Sweden; ^4^ Department of Infectious Diseases University Hospital of Linköping Linköping Sweden

**Keywords:** e‐health, immunodeficiency, nurses, nursing, qualitative, self‐management

## Abstract

**Aim:**

Advances in technology generate new opportunities to develop e‐health tools to help individuals in self‐management by assessing symptoms of illness and its relation to treatments. Self‐management is central when living with primary immunodeficiency diseases. The aim was to explore the experiences of people living with primary immunodeficiency, who used a pilot version of the web‐based health diary.

**Design:**

Explorative design.

**Methods:**

In total, 16 participants (median age 59) attended one of three focus groups. Inductive content analysis was used.

**Results:**

The participants could be encouraged to discover aspects of their health by contributing to documentation which could support the health concept. A greater understanding about their own health and communicating with healthcare professionals during encounters was expressed. The web‐based health diary is a helpful tool to discover aspects of health that affects the individuals’ life situation and assists the self‐management of a long‐term condition such as immunodeficiency.

## 1 INTRODUCTION

The rising prevalence of long‐term conditions is a major challenge facing governments and healthcare systems today (Barnett et al., 2012), which results in an increased demand of improving health care and involving patients in their care (Thompson, 2007). Greater active involvement demanded by patients is necessary to keep up with the realities of long‐term illness, whereby the responsibility for day‐to‐day management gradually shifts from healthcare professionals to the individual patient (Barlow, Wright, Sheasby, Turner, & Hainsworth, 2002). Regarding patients as experts and their ability to access information that is relevant to their health care and to carry out their self‐management tasks at a given point in time is one essential part in future healthcare services (Coulter & Ellins, 2007). Self‐management could be described as the daily process where individuals engage to manage a long‐term condition, which is an interactive and dynamic process, taking place in conjunction with family, healthcare professionals, and community to manage lifestyle changes, treatments, and different consequences of the health condition that may occur (Schulman‐Green et al., 2012). Optimal self‐management entails the ability to monitor the condition and to develop and use behavioural, emotional, and cognitive strategies to maintain a satisfactory quality of life. Self‐management programmes can deliver interventions to facilitate a patient's ability to self‐monitor their health or processes so that they can make informed decisions as active partners in the management of their health (Richard & Shea, 2011). It has been shown that there is a positive effect of such supports. For example, computer‐based interactive health communication applications (e‐health) have a positive effect on users’ knowledge and feelings of support, which may have an effect on improving clinical outcomes and behaviour compared with nonusers (Hanlon et al., 2017; Murray, Burns, See, Lai, & Nazareth, 2005). Advances in technology could generate new opportunities to develop tools to help individuals self‐monitor and assess their symptoms and health status. It has been suggested that applications of such e‐health tools could empower individuals with long‐term conditions to be actively engaged in managing their health (Ovretveit et al., 2013; Wicks, Stamford, Grootenhuis, Haverman, & Ahmed, 2014).

## 2 BACKGROUND

Self‐management is crucial when it comes to primary immunodeficiency (PID), which is a cluster of different congenital lifelong diseases that result in higher rates of recurrent upper and lower respiratory infections such as sinusitis or pneumonia (Vultaggio et al., 2015). The incidence of PID has increased in the last 40 years, likely due to an expanded understanding of the genetic basis and immunological mechanisms in PID. However, the exact prevalence of PID is not known, but it has been estimated that about 1 in 1,200 people in the United States are diagnosed with PID (Boyle & Buckley, 2007). In Sweden, about 40,000 people have been estimated to suffer from PID, but only about 3,000 have been diagnosed so far (SLIPI, 2015). Having a PID diagnosis results in decreased quality of life due to fatigue, severe infections resulting in hospital stays and sick leave and lifelong treatment with replacement therapy of immunoglobulin (Kearns, Kristofek, Bolgar, Seidu, & Kile, 2017; Routes et al., 2016). To follow up achievements in the care provided for patients with PID, a national quality registry (PIDcare) has been developed since 2012. The registry is web based and contains the data of diagnosis, treatments, together with the effects of treatment and the registry has a coverage of more than 80% of all diagnosed patients with PID in Sweden.

As the majority of PID cases are caused by low levels of protective antibodies, one way is to aid self‐management support by monitoring symptoms of infections continuously and to associate the symptoms to decreased level of antibodies and number administered doses of immunoglobulin in a health diary. Paper diaries have been used for a long time in the care of patients with PID, but now the PIDcare registry have opened the up possibility to develop the paper diary in a web‐based form. The web‐based health diary (WHD) consist of eight areas where the patient can register their symptoms (fever, cough, infection signs etc.); use of antibiotics; sick leave days; vaccinations; doses of immunoglobulin treatment; number of inpatient care days; and quality of life measured by the Euroqol‐5 dimensions 5 level questionnaire (EQ‐5D‐5L). The last area is for personal notes, which is not visible for anyone other than the patient themselves. Each item of registered data is transformed into diagrams showing data inserts over time. In light of the knowledge about the benefits concerning self‐management and PID as a long‐term condition, it is of utmost importance to understand more about a patient's own experience of using e‐health tools. The aim of this study was to explore experiences of using a pilot version of the web‐based health diary among people living with primary immunodeficiency.

## 3 METHODS

### 3.1 Design

This pilot study was based on an explorative qualitative design using focus group discussions; to explore patients experiences of using a web‐based health diary (Creswell, 2012).

### 3.2 Participants

Participants were recruited from a university hospital in the south of Sweden that serves about 200 patients with immunodeficiency. To be enrolled in the study, participants (aged at least 18 years) were required to have tested the WHD for at least 1 month and been diagnosed with severe immunodeficiency within the latest year, in addition to accepting to be enrolled to the PIDcare quality registry. A study nurse at the department gave participants an information letter about the pilot study in relation to a clinical visit and then contacted each participant by phone to ask for their consent to participate and to schedule time for interview. A reminder phone call was performed the day before the scheduled interview. In total, 22 participants were asked to participate in the present study, but two declined and four participants were not able to come the day for the interviews due to acute sickness. This left 16 participants divided into three different focus groups with nine females and seven males. Their age ranged between 34‐73 years with a median age at 59 (mean 58). Before the interviews took place, all participants had tested a pilot version of the WHD, which was set up in a testing environment including all functionalities before starting the pilot study. This was made due to the possibility to make improvements in the application before releasing the final version which was completed after this study was performed.

### 3.3 Procedure

All participants attended one of three focus group discussions conducted by an experienced qualitative researcher (CP). Focus group discussions are semistructured discussions with 4–12 participants aiming to explore a specific issue with the intent to encourage participants to share their common experiences. Participants answered questions individually but were encouraged to talk and interact with each other during the interviews (Krueger & Casey, 2015). A predetermined interview schedule was followed to explore experiences from the participants regarding the WHD. The schedule had three content areas and was complemented with probing questions during the interviews. Issues in the content areas covered experiences of using the WHD; how the WHD should be used and about how the WHD could add anything to everyday life functioning. The interviews were recorded on audio tape and one observer made field notes together with a short summary at the end of each interview with the intent of encouraging participants to clarify their statements or to add something that may have been missed during the interviews. Interviews were conducted in a separate room at the clinic and performed by researchers who were unknown to the participants. Each of the focus group discussions lasted approximately 45 min.

### 3.4 Data analysis

Recordings were transcribed verbatim and analysed using inductive qualitative content analysis, which is a technique to make replicable and valid inferences from written texts (Krippendorff, 2013). The analysis followed the outlined steps described by Elo and Kyngäs (2008). After reading through the written texts several times, notes were written that illustrated the essential features of descriptions regarding the experiences of the WHD. Notes were sorted as codes into coding sheets and codes with similarities were separated and grouped into preliminary categories. In the analysis, six subcategories emerged by going back and forth between the preliminary categories and codes. Then, the six subcategories were abstracted into two generic categories, which were based on the underlying meanings of the experiences using the WHD. In the last step, the two generic categories were abstracted into a main category, describing the experiences of using a WHD (Elo & Kyngäs, 2008). To increase credibility, two authors (CP and RF) with experiences of inductive content analysis performed the analysis in an open and critical dialogue. A third author (JB) confirmed the analysis. Trustworthiness was assured by using authentic quotations from all interviews to elucidate each subcategory (Polit & Beck, 2013) and by using an audit trail (Shenton, 2004).

### 3.5 Ethical considerations

The ethical principles of respect for autonomy, nonmaleficence, beneficence, and justice were considered as stated in The Declaration of Helsinki (The Swedish Research Council, 1999). Verbal and written information about the study was given to each participant before entering the interview. The participants gave their informed consent to take part in the study. Based on the nature of the study as a pilot project, approval was given from the operation manager at the clinic. According to Swedish law (SFS 2003:460) the approval from official research committee is not required when conducting a pilot study.

## 4 RESULTS

Findings revealed that using a WHD could encourage participants in discovering aspects of their health, which was described by the two generic categories: “contributing in documentation supports the health concept” and “being knowledgeable of technical potentialities for safety care” (Table 1). When using the WHD, participants could discover aspects of their health when symptoms were visualized in the diagrams. They also had help with the documentation when coming to the clinic, and healthcare professionals not only asked about their health but also asked for more information about the intention of clinical use of the WHD.

**Table 1:** Illustration of the categories that emerged in the analysis

Main category	Generic categories	Subcategories
Discovering aspects of health	Contributing in documentation supports the health concept	Describing health status over time promotes understanding
		Documentation according to personal integrity
		Facilitating communication with professionals
	Being knowledgeable of technical potentialities for safety care	Technical shortcomings
		Unmotivated to use new technology
		Lacking information about usage

### 4.1 Contributing to documentation supports the health concept

Contributing to the documentation about one's own health status in the WHD could motivate patients to be part of the decision‐making process, which was expressed by using the WHD together with the professionals at clinical visits, which in turn could facilitate the communication during encounters. This was illustrated by three subcategories.

#### 4.1.1 Describing health status over time promotes understanding

To use the WHD could be helpful in describing health over time by promoting the understanding about a patient's health status in general and how treatments affect their health. When collecting documentation in the WHD, it was described by participants that it was easy to develop routines to remember to fill out the questions and descriptions of their health: “That overview helps me remember how it was…. You know it's easy to forget when things are getting better…” (group 2). The graphical design of the WHD contributed to an overall picture of health status by the colours, tables, and diagrams. This led to a positive feeling and interest as participants suddenly gained an understanding on how they had been feeling over the past period. Added to this, they could put in own descriptions regarding reports of a symptom caused by a specific situation, for example, having their grandchildren visiting and then catching a cold. By changing the time period in the WHD, they could easily see how they had registered their symptoms months ago, which was another positive aspect that was possible in the WHD comparable to the paper diary: “This is kind of giving me a total picture…. Then I know how it has been...” (Group 1).

#### 4.1.2 Facilitating communication with professionals

Information stored in the WHD could help facilitate communication with professionals. This was experienced as helpful by participants when discussing their symptoms of disease and overall health status with their nurses and physicians. For example, it could be useful when communicating through telephone with the nurse, giving a short overview of past health status and current state. Likewise, the WHD could function as a means to enable patients to be involved in their care, but the professionals need to be prepared by reading the WHD prior to the clinical visit taking place:

I think that we want to be involved no matter the diagnosis…. the time you have with the doctor is short…like…. directed from the beginning….so you want most out of it and then I think that this diary is important but must be read by them before we meet if it supposed to add anything… (Group 1).

Participants also expressed that when meeting the doctor, they often forgot how they had been feeling in past months and sometimes guessed about their past symptoms. By using the WHD, this problem could be eliminated: “I occasionally guess how my symptoms have been during the past months – it's hard to remember how much cough I had back then…” (Group 2).

#### 4.1.3 Documentation according to personal integrity

The documentation according to personal integrity was important and described in terms of options to decide by the participants if they wanted to fill out the WHD or not. Some participants wanted the comments to be visible for the healthcare professionals, but some did not and they agreed that this should be optional:

It could be a free choice…if there was a box to click that this is okay for me to share with my doctor…then you could be active to choose by yourself…if I like to discuss it or not with my doctor…” (Group 3).

If this was an option, the WHD could be used not only in the contact with the health care but to be used as an overall diary for personal use. Some participants had other conditions affecting them and by using the WHD in this certain way, they could describe how other conditions affected their health status. But it was obvious that this needed to be according to the patient's own will to guarantee personal integrity:

I was thinking that I could fill out …like that I am feeling down at the moment and that kind of stuff….and then I know that there will not be someone else that is reading that… (Group 1).

When using the WHD, information was collected in one single place which gave participants a feeling of security, contrasting their experiences of clinical visits where loose paper sheets easily disappeared or were forgotten during the visit.

### 4.2 Being knowledgeable of technical potentialities for safety care

This generic category describes the knowledge and understanding of technology and information regarding the use of the WHD and is important for safety care. The results showed remaining technical shortcomings that needed to be adjusted. Another important aspect was to be informed about how the diary was supposed to be used and understood as a guarantee for safety care. Participants also corresponded about being unmotivated to use the WHD because they lacked interest in technical potentialities in general.

#### 4.2.1 Technical shortcomings

Technical shortcomings were described by participants, since technicalities concerning sharing personal information over a network are complicated due to Swedish law and therefore each participant required their own personal identification login from the authorities. Participants also revealed concerns about the most sick, elderly persons, and how they were supposed to adjust to new technology that obviously was experienced as being complicated. The development of new technology should take into account elderly persons and in the way the WHD was initially developed, it was not that easy to use according to the participants. Another example was the development of free text messages (SMS) which was limited in the tested version. Other shortcomings were problems in using the WHD on several devices. The WHD could be opened on the computer but not on a tablet or smartphone: “I wanted to use my smartphone…. but it only worked on my computer” (Group 2). Other examples that were given by participants included having a reminder as a text message, or automatically registering sick leave days by connections to other electronical healthcare systems. Also, participants noted that registering the batch identification number from their drugs was complicated and suggested that a future version be able to scan this number, as the combination of numbers was difficult to read for someone with visual impairment: “It sure has potential…but now it's a little bit inflexible as I said before…. I also want more space for writing my comments…” (Group 1).

#### 4.2.2 Unmotivated to use new technology

Participants also expressed considerations about being unmotivated to use new technology. For example, having a computer or other devices were not in everyone's interest and it was described as making too many demands of other data systems for privacy issues. Therefore, using the WHD felt difficult to manage and was experienced as just another obligation:

Well…. yes, it depends on how close you are to the computer…. you know…if you don't use it that much it is another step before starting it all and do all the log in stuff…” (group 1).

After testing the WHD, some still favoured the paper diary as they felt that using the paper diary was a faster way instead of the complicated process to log into the system at a computer that they did not use on a regular basis:

I still prefer to insert my health data by hand in the paper diary…. but maybe I will change my mind later…for now I don't think that this was a super hit according to my experience… (group 3).

#### 4.2.3 Lacking in information about usage

The WBD contains questions about general health and participants noted that there was a lack of information about why they were supposed to answer questions that did not mean anything to them. Some questions were described as nonsensical to them because the questions did not describe the essence of their illness. This was the case when participants had other conditions that affected their health more than the immunodeficiency and those questions were asked in a general sense, which easily could be misinterpreted: “My question is the choice of health questionnaire…. Why use EQ5D with those silly questions that don't help to illustrate how I feel concerning my immunodeficiency…” (Group 2). On the other hand, questions about mental health were deemed necessary, but information about how to follow up on this was not described to the participants: “What is the plan at the clinic in using the web‐based diary anyway?........are we going to look at it occasionally……. or only when I come for check‐ups?” (Group 3). It was also expressed by participants that there was a shortage of information on how to interpret the diagrams and the understanding of each field that they were supposed to fill out in the WHD.

## 5 DISCUSSION

Findings in this study revealed experiences that suggest the use of a WHD could promote the discovery of aspects of health by contributing to documentation which could support the health concept. This could be a way to understand more about one's own health together with helping to enable effective communication with healthcare professionals during encounters. The findings also revealed the need to be knowledgeable about the WHD technical potentialities for safety care: This means the need to understand the purpose of the WHD and to be given information about the functionalities as well as an easier to use application.

The main category that emerged was discovering aspects of health that could be helpful in the self‐management of diseases such as immunodeficiency. The overall experience of living with a long‐term condition in general changes over time, which also has an impact on an individual's self‐management. Psychosocial, social, or financial changes can have an impact on self‐management needs, expectations, and routines. The ongoing access to self‐management support and development of expertise regarding this also influences the ability to carry out self‐management processes (Schulman‐Green et al., 2012). As described by Hanlon et al. (2017), the use of e‐health applications is stated as a safe alternative mode of delivery for self‐management support (Hanlon et al., 2017). An understanding of the impact of offering choice or evaluation of other potential benefits of e‐health applications, such as improved health outcomes, needs further attention. If self‐management interventions such as the WHD are intended to have a great uptake by patients, thought must be given to how and when it is offered to patients. The endorsement of such intervention programmes at the clinical visit will probably ensure higher rates of participation (Newman, Steed, & Mulligan, 2004). It should also be recognized that this might not be suitable for all patients. This was obvious in the descriptions from participants who felt that the modern technology in using computers or other electronic devices was difficult, resulting in the lack of interest in using the WHD. Identifying who benefits most from the use of the WHD is an important addition to any assessment and could lead to a more effective way of targeting healthcare recourses (Newman et al., 2004). Training skills for both healthcare professionals that are intended to inform and educate patients as well as for patients during their use of the WHD when obstacles occur is important. If the WHD is to be delivered appropriately and effectively, training skills about using the diary together with clear information about its purpose and use during encounters are essential. Self‐management will vary in importance to a patient's own situation and knowledge and must therefore be regarded in the context of each patient's situation. Healthcare professionals need to be aware of the skills each patient has and how the self‐management activities may affect different situations (Schulman‐Green et al., 2012). Therefore, healthcare professionals should be trained to ensure that patients’ self‐management abilities are maintained and are fostered in the clinical setting. A greater use of education may offer a way forward that is not only built on values and experiences of people with long‐term conditions but may also prove to be cost effective (Barlow et al., 2002).

Contributing to the documentation about one's own health status in the WHD could motivate patients to be part of the decision‐making process, which was expressed by using the WHD together with the professionals who could facilitate the communication during encounters. It is important to build a high‐quality relationship between healthcare professionals and patients, which has been demonstrated by others (McCormack, Karlsson, Dewing, & Lerdal, 2010). When patients are trusted and given the opportunity to decide for themselves where possible, a sympathetic presence and a meaningful engagement in their relationship with the healthcare professionals are likely to occur (McCormack et al., 2010). It has been described that the role played by information technology appears to affect communication, collaboration, and the relationship between patients and nurses in both positive and negative ways. The use of such technology can mediate, support, and intrude on communications, which could be advantageous for patient care in terms of safety and quality (Fagerstrom, Tuvesson, Axelsson, & Nilsson, 2016). Participation relies on the flow of information in two ways; exchanged between the patient and healthcare professionals. This process of dialogue and negotiation could potentially reduce the risk of misinterpretations. As such, participation mechanisms can be incorporated into existing deliberative processes already used in e‐health applications (Facey et al., 2010). Technology devices such as the WHD should be focusing on patient problems and take patients’ perspectives and preferences into considerations. This could empower the patient to develop a sense of ownership in the evaluation of their own health status and consideration of the decision‐making process. This is central in the usage of WHD, as the intent is to ensure that decisions regarding further care are made in partnership between the patient and the healthcare professionals. Indeed, without sufficient involvement from the patient, there is a risk that e‐health applications could be rejected due to organizational pressures and not from a lack of use or adding of value for patients and professionals in clinical encounters (Facey et al., 2010).

Participants expressed a desire to be informed about how the diary was supposed to be used and this was another important aspect to be understood as a guarantee for safety care. If new technology devices such as a WHD will be used in clinical practice settings, it is important to target the shortcomings of information technology services to better and more effectively meet demands for security, quality, and efficiency. Putting information technology and such devices as the WHD on the agenda at different levels in the healthcare organization, could help to potentially exploit the full potential of this technology as a part of a modern healthcare system. Furthermore, the quality of the deliberative process of participation relies on a patient's ability to contribute competently, which must be established on training: that is, knowledge about technicalities about the e‐health application. This should be built on a foundation of mutual respect and opportunity for participation with the ability to opt out (Facey et al., 2010). The analysis of effectiveness and safety in the use of e‐health applications demonstrate that users on one hand are valuing convenience and may express a sense of being watched over and on the other hand may feel empowered using such interventions (Hanlon et al., 2017).

### 5.1 Methodological considerations

There are several limitations to this study that should be borne in mind when interpreting the results. First, the sample size was relatively small, but was deemed sufficient according to the nature of a pilot study. Given the nature of an explorative qualitative design, the generalization of study results is limited, but results can shed light on considerations in the future development of e‐health applications and about its use in clinical practice settings. The location of the focus group discussions may have affected the results, since participants may have been less willing to provide negative statements about the WHD. When using qualitative inquiry, trustworthiness should be discussed using the concepts of credibility, dependability, conformability, and transferability (Lincoln & Guba, 1985). In this study, credibility was assured by debriefing sessions between the authors during analysis and dependability was established by describing the analysis process in detail. Conformability was assured by using authentic quotations from all focus group interviews. Transferability could be seen in the light of describing participants and settings but is limited in this study due to the design of the study (Elo et al., 2014).

### 5.2 Implications for practice

The knowledge from this pilot study may guide the future development of e‐health applications aiming to provide self‐management support to people living with long‐term health conditions. It is also important to ask those who are affected, since participants in this study claimed more information about the purpose of the WHD and how it was supposed to be used in a clinical practice setting. The coproduction of services should be developed and is an important ingredient in future improvements of the healthcare system. This is inevitable if healthcare should be built on the participation and engagement from a patient's point of view.

## 6 CONCLUSIONS

To be able to self‐manage a long‐term condition such as immunodeficiency, the WHD is a helpful tool in discovering aspects of health that affect an individuals’ life situation. The WHD could extend its use if healthcare professionals incorporate the WHD into daily clinical praxis and are prepared by reading the WHD before meeting the patient at the clinic. Each person has different prejudices concerning the use of modern technology and therefore, the use of self‐management tools for e‐health such as the WHD must be voluntary and based on each patient's individual needs. Future research should focus on how the clinical encounter could be directed when using e‐health tools for self‐management for patients with long‐term conditions.

## CONFLICT OF INTEREST

The authors declare no conflict of interest. The authors alone are responsible for the content and writing of this paper.

## AUTHOR CONTRIBUTIONS

Christina Petersson and Janne Björkander contributed to the design of the study. Data were selected by Christina Petersson and Ramona Fust. All of the authors drafted the manuscript, took part in the analysis process, and made critical revisions.
